# Does culture shape hunting behavior in bonobos?

**DOI:** 10.7554/eLife.62104

**Published:** 2020-09-01

**Authors:** Andrew Whiten

**Affiliations:** School of Psychology, University of St AndrewsSt AndrewsUnited Kingdom

**Keywords:** Pan paniscus, hunting, group specific, intergroup dynamics, culture, social learning, Other

## Abstract

New evidence that neighboring communities of bonobos hunt different prey species, despite extensive overlaps in where they live and hunt, is difficult to explain without invoking cultural factors.

**Related research article** Samuni L, Wegdell F, Surbeck M. 2020. Behavioral diversity of bonobo prey preferences as a potential cultural trait. *eLife* **9**:e59191. doi: 10.7554/eLife.59191

Culture, defined as any group-specific behavioral patterns that are acquired by learning from others (Laland and Janik, 2006), has been identified in a wide range of animal species, spanning all vertebrate groups and likely including invertebrates such as insects (Whiten, 2017). The transmission of behavior via such social learning may also extend across generations to provide a secondary form of behavioral inheritance, in addition to whatever genetic inheritance achieves (Whiten, 2017). If confirmed, this could be important for a full understanding of behavioral evolution.

Research on our closest animal relative, the chimpanzee *Pan troglodytes*, has long been at the forefront of this work, since decades of fieldwork began to reveal multiple behavioral variations between communities studied across Africa, including forms of tool use, grooming patterns and social behaviors (McGrew, 1992). In this species culture has been investigated through a combination of observational, statistical and experimental methodologies, and it is now clear that chimpanzees have the capacity to transmit multiple traditions through social learning (Whiten, 2017). Cultures encompassing multiple different traditions have also been identified in the other genera of great apes, gorillas and orangutans.

There is, however, a ‘poor relation’ in all this scientific endeavor: culture has been only tentatively explored in the wild in the sister species of the chimpanzee, the bonobo *Pan paniscus*, (Hohmann and Fruth, 2003). This is for a number of reasons, the simplest of which is that bonobos live only in the inner Congo Basin of the often war-torn Democratic Republic of the Congo and few bonobo communities have been habituated and studied. Now, in eLife, Liran Samuni, Franziska Wegdell and Martin Surbeck report the results of a study that begins to fill this cultural lacuna for bonobos (Samuni et al., 2020).

The study capitalizes on a characteristic of this species – that neighboring communities of bonobos are very tolerant of each other. Samuni et al. were able to show that despite a massive 65% overlap in the home and hunting ranges of two neighboring groups of bonobos – named the Ekalakala and Kokoalongo – they pursued and consumed different species of prey. Members of the Ekalakala group displayed a focus on anomalures (a large, flying-squirrel-like rodent) to the almost total exclusion of squirrels and duikers (a type of antelope), whereas the Kokoalongo group had exactly the reverse preferences (Figure 1).

**Figure 1.**
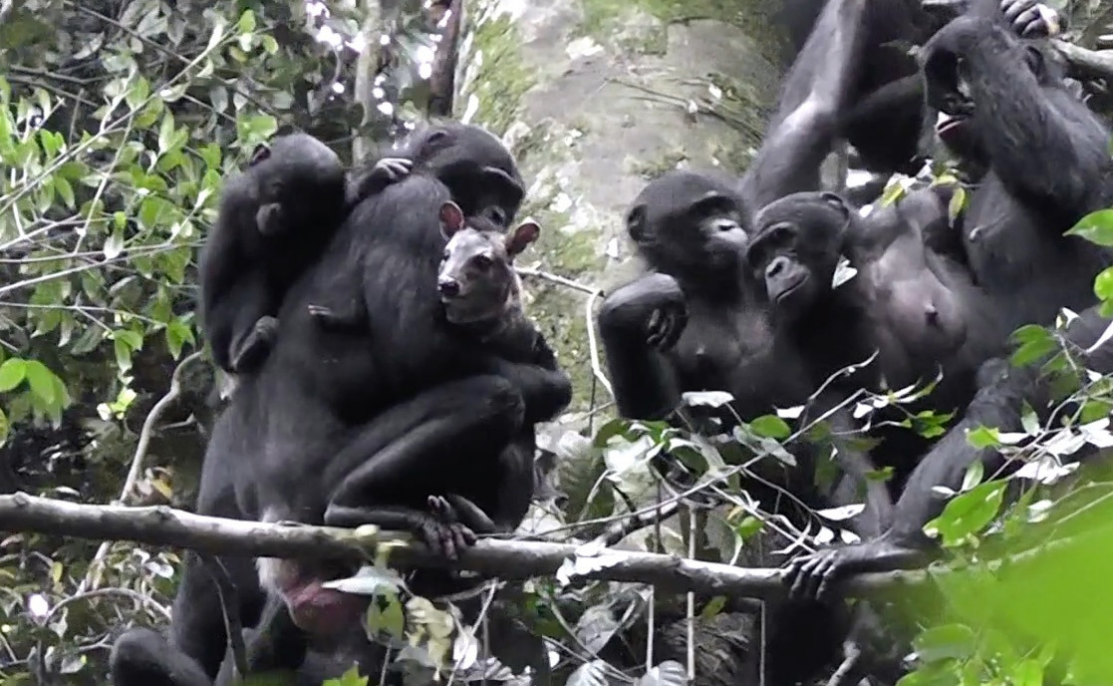
Hunting behavior and prey preference in bonobos. Samuni et al. studied two neighboring groups of bonobos – the Ekalakala and the Kokoalongo – with overlapping home and hunting ranges. However, despite these overlaps, and multiple encounters between the two groups, members of the Ekalakala group almost exclusively hunted for anomalures, whereas members of the Kokoalongo group mostly hunted for squirrels and duikers. This still, from a video recorded by Samuni et al., shows a member of the Kokoalongo group holding a duiker it has captured. Samuni et al. suggest that the difference in prey preferences between the two groups is due to cultural factors.

Studies of chimpanzees have identified similar behavioral differences between neighboring communities, as has at least one other primate study (on vervet monkeys: Tournier et al., 2014). In the Taï Forest of Cote d’Ivoire, nut-cracking chimpanzees in neighboring communities display different variants of certain behaviors, notably in their seasonal preferences for stone versus wooden materials for cracking nuts (Luncz and Boesch, 2014). Similarly, at Gombe in Tanzania, neighboring communities differ in the width, length and variety of their termite fishing probes (Pascual-Garrido, 2019). And in the Budongo Forest of Uganda, neighboring chimpanzee communities display differing prey preferences (Hobaiter et al., 2017).

Studies of neighboring communities can more strongly reject environmental or genetic variation as potential explanations for cultural differences than can studies that compare widely separated communities. However, chimpanzees are extremely territorial, displaying lethal aggression against their neighbors. This tends to minimize overlaps between communities, so the worry lingers that some subtle habitat difference that has not yet been observed may instead explain the behavioral variations. By contrast, 80% of the bonobo hunts reported by Samuni et al. occurred in the overlap zone of the two groups, so they shared the same hunting and prey opportunities. Despite this, they showed the dramatic differences in prey preferences described above. Samuni et al. accordingly conclude that these are indeed cultural variations.

This discovery is the more striking because in addition to the extensive overlap zone, the social tolerance of the bonobos extends to long periods of social association, during which a mixture of affiliative and aggressive encounters take place. One might expect these tolerant associations to corrupt any behavioral differences between the groups through opportunities for social learning. The fact that this does not occur suggests, instead, a marked conformity to the behavioral norms of one’s own group. In the study of nut-cracking chimpanzees mentioned above it was shown that females who had transferred from one community to another conformed to the preferences of their new community (Luncz and Boesch, 2014): it would be fascinating to discover if the same happened in bonobos.

Such conformity might be adaptive if it supports different groups using different techniques to hunt for different types of prey in the same habitat (in much the same way that different species exploit different ecological niches to survive in the same habitat). Samuni et al. – who are based at Harvard University, the Bonobo Conservation Initiative and the Max Planck Institute of Evolutionary Anthropology – refer to this hypothesis as ‘micro-level niche differentiation’. This is reminiscent of the different ‘ecotypes’ that occur among populations of killer whales, which are thought to be culturally-based. Some populations hunt seals, others fish, with each specialization requiring the social learning and mastery of the particular highly skilled techniques they witness around them (Foote et al., 2016). It would also be fascinating to know if such cultural foraging niche separation occurs amongst human hunter gatherers if extensive range overlaps exist between communities.

Coincidentally, a recent study from a very different perspective offers further evidence correcting the paucity of data on bonobo culture (van Leeuwen et al., 2020). This work, which studied populations of bonobos at six zoos, suggests that two social behavior patterns (‘social scratch’ and ‘groom slap’) have been culturally transmitted within these groups. Accordingly the two new studies, one in the wild, the other in captivity, studies that use entirely different methodologies to identify culture in hunting behavior and in social behavior respectively, at last put bonobos seriously on the ape cultural map.
